# Clinical and genetic features of lung squamous cell cancer in never-smokers

**DOI:** 10.18632/oncotarget.8745

**Published:** 2016-04-15

**Authors:** Yangle Huang, Rui Wang, Yunjian Pan, Yang Zhang, Hang Li, Chao Cheng, Difan Zheng, Shanbo Zheng, Yuan Li, Xuxia Shen, Haichuan Hu, Deng Cai, Shengfei Wang, Yawei Zhang, Jiaqing Xiang, Yihua Sun, Jie Zhang, Haiquan Chen

**Affiliations:** ^1^ Department of Thoracic Surgery, Fudan University Shanghai Cancer Center, Shanghai, China; ^2^ Department of Thoracic Surgery, Shanghai Chest Hospital, Shanghai Jiaotong University, Shanghai, China

**Keywords:** squamous cell lung cancer, never-smoker, clinicopathological characteristics, genetic features

## Abstract

To evaluate the importance of specific driver mutations to the development and outcome of lung squamous cell cancer (SQCC) in never-smokers, we assessed the clinicopathological characteristics and outcomes of 597 patients who underwent complete resection of SQCCs. In total, 88 (14.7%) never-smokers and 509 (85.3%) ever-smokers were compared. The never-smokers included more females (42.05% vs. 1.57%, *P* < 0.001) and more often had a personal history of malignant disease (9.09% vs. 2.36%, *P* = 0.003). The tumors of never-smokers were more often poorly differentiated (70.45% vs. 53.24%, *P* = 0.010) and more often contained oncogenic mutations (21.05% vs 11.05%, *P* = 0.023), particularly *EGFR* mutations (13.16% vs 3.40%, *P* = 0.001). Never-smokers also tended to have poorer OS than smokers. Our results suggest lung SQCCs in never-smokers are a subtype distinct from SQCCs occurring in smokers.

## INTRODUCTION

Lung cancer remains the leading cause of cancer-related deaths worldwide, accounting for over 1 million deaths yearly [1]. Approximately, 85% of newly diagnosed lung cancers are non-small cell lung cancers (NSCLCs). Its two major histological types, adenocarcinoma (ADC) and squamous cell carcinoma (SQCC), account for 30– 50% and 20–30% of NSCLC, respectively [2]. Tobacco smoking has long been recognized as a major risk factor for lung cancers. However, approximately 25% of lung cancer patients are lifelong never-smokers, which means that lung cancer in never-smokers would rank as the seventh most common cause of cancer mortality around the world, if considered as a separate category [3].

Several etiologic factors have been proposed to explain the development of lung cancer in never-smokers [4]. Among them are driver mutations within genes encoding signaling proteins critical for both the initiation and maintenance of lung malignancy, which have been identified in a series of molecular and translational studies [5]. Lung ADC and SQCC exhibit distinct genetic mutation profiles that underlie their differing responses to targeted therapies. Previous studies have shown that *EGFR*, *KRAS*, *HER2* and *BRAF* contain recurrent driver mutations in ADC [6–8] and that these mutations are seen particularly frequently in females, never-smokers and Asian patients [9–11].

The aforementioned mutations also occur in SQCCs [12], though few studies have focused specifically on SQCC among never-smokers, and the clinicopathological characteristics and genetic features of this subgroup are not well established. We were interested in whether SQCCs in never-smokers harbor the known *EGFR* and *KRAS* driver mutations. We therefore characterized the features of this subgroup to determine whether SQCC in never-smokers is distinct entity from that in ever-smokers. Our findings provide important information that could be useful for selecting patients with specific driver mutations for future clinical trials of individualized therapies for SQCC.

## RESULTS

### Clinicopathological characteristics

A total of 597 patients met the enrollment criteria and were entered into our study cohort, which including 88 (14.7%) never-smokers and 509 (85.3%) ever-smokers. All the patients were Chinese and were diagnosed as lung SQCC based on postoperative pathological reports. There were 552 (92.5%) males and 45 (7.5%) females, with an average age of 61.46 years and ages ranging from 36.33 to 87.83 years. Details of clinicopathological features of the never-smokers and their SQCCs are summarized in Table 1, and a comparison of the clinicopathological features of never-smokers and ever-smokers is shown in Table 2. The never-smokers had a considerably higher ratio of female patients than the ever-smokers (42.05% vs. 1.57%, *P* < 0.001). Compared to those in ever-smokers, SQCCs in never-smokers positively correlated with a personal history of malignant disease (9.09% vs. 2.36%, *P* = 0.003) and showed poor differentiation (70.45% vs. 53.24%, *P* = 0.010), and were negatively correlated with a family history of malignant disease (10.23% vs. 24.36%, *P* = 0.003). There were no statistically significant differences between the two groups with respect to age, central/peripheral type, pTNM stage or maximum length of tumor.

**Table 1:** Clinicopathological features of SQCCs in never-smokers

Value	Frequency	Percentage
Gender		
Male	51	58.0%
Female	37	42.0%
Age		
< 55	21	23.9%
≥ 55	67	76.1%
Mean (years) ± SD	61.39 ± 10.40	
Personal History of Malignant Tumor		
Yes	8	9.1%
No	80	90.9%
Family History of Malignant Tumor		
Yes	9	10.2%
No	79	89.8%
Locations of tumor		
peripheral	34	38.6%
central	54	61.4%
N-stage		
0	45	51.1%
1	16	18.2%
2	26	29.5%
3	1	1.1%
Differentiated Degree		
well & moderate	25	28.4%
poor	62	70.5%
unknown &	1	1.1%
Maximum length of tumor		
< 3	15	17.0%
≥ 3	70	79.6%
unavailable!	3	3.4%
Mean (cm) ± SD	4.43 ± 2.28	
Methods of surgery		
pneumonectomy	9	10.2%
lobectomy*	75	85.2%
others^	4	4.5%
Pathological stage		
I	34	38.6%
II	20	22.7%
III	31	35.2%
IV	3	3.4%
oncogenic mutations		
present’	16	18.2%
absent	60	68.2%
unknown	12	13.6%

SD: standard deviation;

& definite degree of differentiation was not determined for 1 patient.

! could not be determined from the final pathological reports.

* including 7 patients who received sleeve resection.

^ 3 patients received wedge resection, 1 patient received sublobar resection.

§ including EGFR, KRAS, HER2, BRAF, AKT1, PIK3CA and FGFR.

**Table 2:** Comparison of the clinicopathological and molecular features of never- and ever-smokers

Value	Never-smokers (*N* = 88)		Ever-smokers (*N* = 509)		*P*
	Freg	Per	Freg	Per	
Gender					
Male	51	57.95%	501	98.43%	< 0.001*
Female	37	42.05%	8	1.57%	
Age					
< 55 years old	21	23.86%	94	18.47%	0.243*
≥ 55 years old	67	76.14%	415	81.53%	
mean ± SD (years old)	61.39 ± 10.40		61.47 ± 8.31		0.945#
Personal History of Malignant Diseases					
No	80	90.91%	497	97.64%	0.003*
Yes	8	9.09%	12	2.36%	
Family History of Malignant Diseases					
No	79	89.77%	385	75.64%	0.003*
Yes	9	10.23%	124	24.36%	
Locations of tumor					
peripheral	34	38.64%	164	32.22%	0.238*
central	54	61.36%	345	67.78%	
N-Stage					
0	45	51.14%	295	57.96%	0.153§
1	16	18.18%	95	18.66%	
2	26	29.55%	118	23.18%	
3	1	1.14%	1	0.20%	
Differentiated Degree					
well & moderate	25	28.41%	230	45.19%	0.01*
poor	62	70.45%	271	53.24%	
unknown	1	1.14%	8	1.57%	
Maximum length of tumor					
< 3 cm	15	17.05%	111	21.60%	0.081*
≥ 3 cm	70	79.55%	394	76.65%	
unknown	3	3.41%	9	1.75%	
mean ± SD (cm)	4.43 ± 2.28		4.38 ± 2.18		0.844 #
Pathological stage					
0	0	0.00%	1	0.20%	0.087§
I	34	38.64%	219	43.03%	
II	20	22.73%	150	29.47%	
III	31	35.23%	138	27.11%	
IV	3	3.41%	1	0.20%	
oncogenic mutations					
absent	60	78.95%	314	88.95%	0.023*
present	16	21.05%	39	11.05%	
unknown	12		156		

SD: standard deviation;

* Pearson's chi-squared test or Fisher's exact.

# Student *t*-test.

§ Mann-Whitney *U* test.

### Spectra of mutations

**Figure 1:**
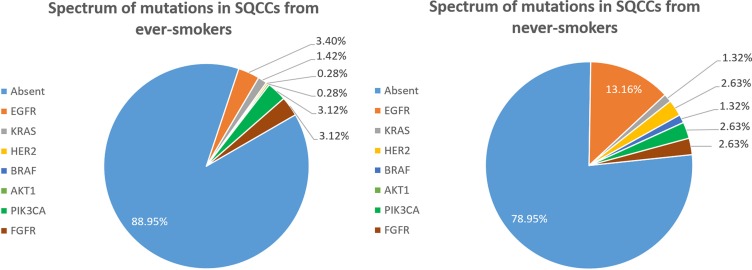
Spectrum of mutations in SQCCs from never-smokers (A) and ever-smokers (B)

The cDNA samples from 429 patients were sufficient and of high enough quality to detect oncogenic mutations. Among the 76 never-smokers, 16 harbored known oncogenic mutations, including 10 (13.16%) *EGFR*, 1 (1.32%) *KRAS*, 2 (2.63%) *HER2*, 1 (1.32%) *BRAF*, 2 (2.63%) *PIK3CA* and 2 (2.63%) *FGFR* fusion. No *AKT1* mutations were detected. One patient harbored both *EGFR* and *PIK3CA* mutations, and another patient had both *EGFR* and *FGFR* mutations. Among 353 ever-smokers, 39 oncogenic mutations were detected, including 12 (3.40%) *EGFR*, 5 (1.42%) *KRAS*, 1 (0.28%) *HER2*, 1 (0.28%) *AKT1*, 11 (3.12%) *PIK3CA* and 11 (3.12%) *FGFR* fusion. No *BRAF* mutations were detected in ever-smokers. One patient harbored both *EGFR* and *PIK3CA* mutations, and another patient had both *KRAS* and *PIK3CA* mutations. Figure 1 illustrates the spectra of detected mutations in the two groups. As shown in Table 2, SQCCs in never-smokers harbored significantly more oncogenic mutations than those in ever-smokers (21.05% vs. 11.05%, *P* = 0.023), especially *EGFR* mutations (13.16% vs. 3.40%, *P* = 0.001).

### Correlations between clinicopathological characteristics and EGFR mutations in SQCCs from never-smokers

Among the 76 SQCCs from never-smokers, there were no statistically significant correlations with respect to gender, age, history of malignant diseases, tumor location, smoking status, pTNM stage or tumor differentiation between patients with *EGFR* wild-type and those with the mutant gene (Table 3).

**Table 3:** Correlation between clinicopathological features and mutational status in SQCCs from never-smokers

Value	EGFR/KRAS – (*N* = 66)		EGFR/KRAS + (*N* = 10)		*P* value
	Freq	Per	Freq	Per	
Gender					
Male	39	59.09%	4	40.00%	0.428*
Female	27	40.91%	6	60.00%	
Age					
< 55 years old	16	24.24%	3	30.00%	0.704*
≥ 55 years old	50	75.76%	7	70.00%	
mean ± SD (years old)	61.88 ± 1.30		58.49 ± 2.97		0.34#
Personal History of Malignant diseases					
No	59	89.39%	10	100.00%	0.584*
Yes	7	10.61%	0	0.00%	
Family History of Malignant diseases					
No	61	92.42%	8	80.00%	0.497*
Yes	5	7.58%	2	20.00%	
Locations of tumor					
peripheral	24	36.36%	7	70.00%	0.095*
central	42	63.64%	3	30.00%	
N-Stage					
0	33	50.00%	5	50.00%	0.847§
1	12	18.18%	1	10.00%	
2	20	30.30%	4	40.00%	
3	1	1.52%	0	0.00%	
Differentiated Degree					
well & moderate	18	27.27%	2	20.00%	0.919*
poor	48	72.73%	8	80.00%	
Maximum length of tumor					
< 3 cm	11	17.74%	2	12.50%	0.369*
≥ 3 cm	53	85.48%	7	43.75%	
unknown	2	3.23%	1	6.25%	
mean ± SD (cm)	4.52 ± 0.29		3.48 ± 0.64		0.191#
Pathological stage					
I	24	38.71%	5	31.25%	0.756§
II	15	24.19%	1	6.25%	
II	25	40.32%	3	18.75%	
IV	2	3.23%	1	6.25%	

WT: wild type; MT; mutant type; SD: standard deviation;

* Pearson's chi-squared test or Fisher's exact.

# Student's *t*-test.

§ Mann–Whitney *U* test.

### Clinical outcomes

**Figure 2:**
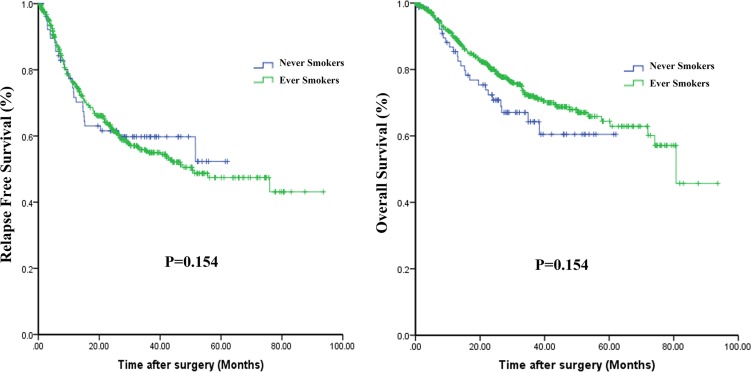
Kaplan-Meier curves comparing relapse-free survival (A) and overall survival (B) among never-smokers and ever-smokers

Survival data were available from 544 patients with SQCC. Kaplan-Meier curves comparing RFS and OS in never-smokers and ever-smokers are shown in Figure 2. No statistically significant difference in RFS (*P* = 0.846) or OS (*P* = 0.155) was found between the two groups. However, there was tendency for OS to be poorer among never-smokers than ever-smokers.

## DISCUSSION

Thanks to major progress in translational medicine, we can now define lung cancers at the molecular level, and distinct treatments can be applied to different disease subtypes. For lung ADCs, driver mutations to *EGFR*, *KRAS*, *HER2*, *BRAF* and *AKT1* have been well reported [6, 13–15], and considerable progress has been made in their treatment [16]. However, whether lung SQCCs harbor these known driver mutations remains controversial [17, 18]. Lung SQCCs are strongly associated with tobacco exposure and are characterized by a high mutational burden with a mean somatic mutation rate of 8.1 mutations per megabase [19]. Despite the high rate of somatic mutations in SQCC, many of them are “passenger” mutations, which are biologically neutral and do not confer a growth advantage [20]. That is why the targeted agents developed for ADC are largely ineffective against SQCC.

Our observation that 14.7% (95% CI, 12.1%–17.4%) of participants in this study were never-smokers suggests lung SQCC is no longer solely a smoker's disease. Furthermore, our comparison the clinicopathological characteristics of SQCCs and the profiles of major known oncogenic mutations revealed that SQCCs arising in never-smokers distinctly differ from those in the more common smoking-related SQCCs. Oncogenic mutations, especially *EGFR* mutations, occurred considerably more frequently in the never-smokers, which is consistent with findings from an earlier study [18].

Controversy remains as to whether lung SQCCs actually harbor *EGFR* mutations. In a study where lung SQCCs were identified based biomarker expression, *EGFR* mutations were absent, suggesting SQCCs found to contain *EGFR* mutations were actually misdiagnosed poorly differentiated ADCs or adenosquamous carcinoma [21]. A previous study from our institution demonstrated that *EGFR* mutations were extremely rare or absent in patients with pure SQCC, whereas *EGFR* mutations occurred frequently in SQCCs with a minor glandular component (SQCC-mGC) [22], and most SQCC-mGCs were diagnosed in never-smokers. That said, other studies showed that *EGFR* mutations were also present in pure SQCCs, especially in females and never-smokers [23]. Moreover, a study by Kang et al. showed that *EGFR* mutations were present in both adenocarcinomatous and squamous cell carcinomatous components of tumors, and were more commonly detected in never-smokers [24]. In our present study, 7 of 10 (70.0%) SQCCs from never-smokers carried *EGFR* mutations, and 7 of 12 (58.3%) SQCCs with *EGFR* mutations from ever-smokers had minor glandular components. This suggests a relation between the glandular component and EGFR mutations in lung SQCCs. Given the substantial inter- and intratumoral heterogeneity of NSCLCs [25, 26], full elucidation of distinct differences between the histogenesis and molecular pathogenesis of SQCCs in never- and ever-smokers will require further study.

In our research, never-smokers with SQCCs tended to have a worse OS than ever-smokers. This may in part be attributable to the poorer tumor differentiation in the never-smokers, though the effect did not reach statistical significance, likely due to insufficient sample size. Consistent with our findings, a recent Japanese study showed that never-smokers with SQCC tended to have a worse prognosis than smokers [28]. Notably, those investigators also found that the prognosis in a light smoking group was significantly poorer than that of heavy smokers. Although the risk of tobacco exposure for lung cancer has been well established for decades [29], the aforementioned results with never-smokers indicate there are factors other than tobacco carcinogens that influence the development of SQCC, which may account for the seemingly contrary prognostic tendency. It is also important to consider the effect of passive smoking in the home, workplace or public on never-smokers. However, the impact of passive smoking on the incidence of SQCCs and *EGFR* mutations in never-smokers is not yet clear [30, 31].

In our study, two patients with *EGFR* mutations were treated with *EGFR*-TKIs, but the efficacy is unclear. Consequently, determination of whether *EGFR*-TKIs are a useful treatment for SQCCs with *EGFR* mutations in never-smokers will require further study.

In sum, our study revealed SQCCs in never-smokers to be a distinct subtype that occurs disproportionally in females and is characterized by poor tumor differentiation and relatively high frequency of *EGFR* mutations along with other known oncogenic mutations. These features may help to identify patients with specific driver mutations for future clinical trials of individualized therapies for lung SQCC.

## MATERIALS AND METHODS

### Patients and samples

From October 2007 to April 2013, we performed consecutive lung tumor resections with curative intent at our institution, the Department of Thoracic Surgery, Fudan University Shanghai Cancer Center. Tumor tissues were prospectively sampled immediately after surgical resections. Samples eligible for this study were all pathologically confirmed SQCC. Morphologic examinations were performed by at least two independent pathologists, and samples were classified according to the new World Health Organization (WHO) classification. Upon immunohistochemical staining for ΔNp63 and TTF-1, a ΔNp63-diffuse/TTF-1-negative profile supported SQCC, while double-positive staining in distinct cell populations supported bi-phenotypic differentiation (adenosquamous carcinoma). Patients who had other malignant diseases within 5 years before surgery were excluded, unless pathologically confirmed as primary lung cancers by experts. Those with a history of occupational exposure to asbestos or other heavy industrial pollutants were also excluded. “Never-smokers” referred to individuals who had smoked less than 100 cigarettes during their lifetime, while “ever-smokers” included those who were current or former smokers. This research was approved by the Institutional Review Board of the Fudan University Shanghai Cancer Center, Shanghai, China. All patients in our study signed written informed consent forms.

### Clinicopathological characteristics

Patients’ clinical characteristics were retrieved from the medical records. Clinicopathological data collected for analyses included gender, age at diagnosis, smoking status, location of tumor, history of other malignant diseases, pathological TNM, tumor differentiation and family history of cancer in first-degree relatives (parents, siblings and offspring). TNM stages were evaluated in accordance with the seventh edition of the lung cancer staging classification system (International Association for the Study of Lung Cancer) [13]. The dates of disease relapse and death were obtained from the clinical records or by telephone.

### Mutational analysis

RNA was extracted from the frozen samples using TRIZOL (Invitrogen) according to the standard protocols (RNeasy Mini Kit; Qiagen, Hilden, Germany) and then reverse transcribed into cDNA using a Revert Aid First Strand cDNA Synthesis Kit (Fermentas, St Leon-Rot, Germany). *EGFR* (exons 18–22), *KRAS* (exons 2–3), *HER2* (exons 18–21), *BRAF* (exons 11–15), *AKT1* (exons 2–3), *PIK3CA* (exons 9 and 20) and 14 known *FGFR* gene fusions were amplified using polymerase chain reaction (PCR) with KOD Plus Neo DNA polymerase (Toyobo Co, Ltd) and samples of cDNA. The PCR was performed in a 25-mL reaction tube on a Mastercycler pro PCR apparatus (Eppendorf AG). RNase-free water was used as a PCR-negative control. The amplified products were analyzed by direct dideoxynucleoside sequencing, after which Chromas Lite, version 2.4 (Technelysium Pty Ltd) and Vector NTI 11.0 (Life Technologies Corp) were used to detect mutations through comparison with standard gene sequences, as previous reported [22].

### Statistical methods

Associations between two categorical variables were analyzed using Pearson's chi-squared test or Fisher's exact test, as appropriate. Continuous and ranked variables were compared using independent sample *t*-tests and Mann-Whitney *U* tests, respectively. Relapse-free survival (RFS) was measured as the interval from the date of surgery to initial tumor relapse (local recurrence or distant) or death from any causes or the last date the patient was known to be alive. Overall survival (OS) was calculated from the time of surgery to death or last follow-up date. Survival curves were constructed using the Kaplan-Meier method. Differences between the curves were evaluated using the log-rank test. Statistical analyses were performed using SPSS for Windows version 19.0 (IBM Corporation, Armonk, NY, USA). All tests were two-tailed, and values of *P* < 0.05 were considered significant.

## References

[1] Siegel R, Ma J, Zou Z, Jemal A (2014). Cancer statistics, 2014. CA Cancer J Clin.

[2] Gibbs AR, Thunnissen FB (2001). Histological typing of lung and pleural tumours: third edition. Journal of clinical pathology.

[3] Sun S, Schiller JH, Gazdar AF (2007). Lung cancer in never smokers—a different disease. Nature reviews Cancer.

[4] Subramanian J, Govindan R (2007). Lung cancer in never smokers: a review. Journal of clinical oncology.

[5] Bronte G, Rizzo S, La Paglia L, Adamo V, Siragusa S, Ficorella C, Santini D, Bazan V, Colucci G, Gebbia N, Russo A (2010). Driver mutations and differential sensitivity to targeted therapies: a new approach to the treatment of lung adenocarcinoma. Cancer treatment reviews.

[6] Sun Y, Ren Y, Fang Z, Li C, Fang R, Gao B, Han X, Tian W, Pao W, Chen H, Ji H (2010). Lung adenocarcinoma from East Asian never-smokers is a disease largely defined by targetable oncogenic mutant kinases. Journal of clinical oncology.

[7] Zhang Y, Sun Y, Pan Y, Li C, Shen L, Li Y, Luo X, Ye T, Wang R, Hu H, Li H, Wang L, Pao W (2012). Frequency of driver mutations in lung adenocarcinoma from female never-smokers varies with histologic subtypes and age at diagnosis. Clinical cancer research.

[8] Zheng D, Wang R, Pan Y, Zheng S, Zhang Y, Li H, Cheng C, Gong R, Li Y, Shen X, Hu H, Cai D, Cheng X (2015). Prevalence and Clinicopathological Characteristics of BRAF Mutations in Chinese Patients with Lung Adenocarcinoma. Ann Surg Oncol.

[9] Shigematsu H, Lin L, Takahashi T, Nomura M, Suzuki M, Wistuba II, Fong KM, Lee H, Toyooka S, Shimizu N, Fujisawa T, Feng Z (2005). Clinical and biological features associated with epidermal growth factor receptor gene mutations in lung cancers. Journal of the National Cancer Institute.

[10] Stephens P, Hunter C, Bignell G, Edkins S, Davies H, Teague J, Stevens C, O'Meara S, Smith R, Parker A, Barthorpe A, Blow M, Brackenbury L (2004). Lung cancer: intragenic ERBB2 kinase mutations in tumours. Nature.

[11] Shigematsu H, Takahashi T, Nomura M, Majmudar K, Suzuki M, Lee H, Wistuba II, Fong KM, Toyooka S, Shimizu N, Fujisawa T, Minna JD (2005). Somatic mutations of the HER2 kinase domain in lung adenocarcinomas. Cancer research.

[12] Qiong Z, Na WY, Bo W, Zhu Z, Ling P, Bo MH, Min TY, Lei Z, Na HD, Bo Z, Fang LJ, Seng ZS (2015). Alterations of a spectrum of driver genes in female Chinese patients with advanced or metastatic squamous cell carcinoma of the lung. Lung cancer.

[13] Pao W, Girard N (2011). New driver mutations in non-small-cell lung cancer. The Lancet Oncology.

[14] Li C, Fang R, Sun Y, Han X, Li F, Gao B, Iafrate AJ, Liu XY, Pao W, Chen H, Ji H (2011). Spectrum of oncogenic driver mutations in lung adenocarcinomas from East Asian never smokers. PloS one.

[15] Gao B, Sun Y, Zhang J, Ren Y, Fang R, Han X, Shen L, Liu XY, Pao W, Chen H, Ji H (2010). Spectrum of LKB1, EGFR, and KRAS mutations in chinese lung adenocarcinomas. Journal of thoracic oncology.

[16] Mok TS, Wu YL, Thongprasert S, Yang CH, Chu DT, Saijo N, Sunpaweravong P, Han B, Margono B, Ichinose Y, Nishiwaki Y, Ohe Y, Yang JJ (2009). Gefitinib or carboplatin-paclitaxel in pulmonary adenocarcinoma. The New England journal of medicine.

[17] Ohtsuka K, Ohnishi H, Fujiwara M, Kishino T, Matsushima S, Furuyashiki G, Takei H, Koshiishi Y, Goya T, Watanabe T (2007). Abnormalities of epidermal growth factor receptor in lung squamous-cell carcinomas, adenosquamous carcinomas, and large-cell carcinomas: tyrosine kinase domain mutations are not rare in tumors with an adenocarcinoma component. Cancer.

[18] Miyamae Y, Shimizu K, Hirato J, Araki T, Tanaka K, Ogawa H, Kakegawa S, Sugano M, Nakano T, Mitani Y, Kaira K, Takeyoshi I (2011). Significance of epidermal growth factor receptor gene mutations in squamous cell lung carcinoma. Oncol Rep.

[19] Cancer Genome Atlas Research N (2012). Comprehensive genomic characterization of squamous cell lung cancers. Nature.

[20] Kan Z, Jaiswal BS, Stinson J, Janakiraman V, Bhatt D, Stern HM, Yue P, Haverty PM, Bourgon R, Zheng J, Moorhead M, Chaudhuri S, Tomsho LP (2010). Diverse somatic mutation patterns and pathway alterations in human cancers. Nature.

[21] Rekhtman N, Paik PK, Arcila ME, Tafe LJ, Oxnard GR, Moreira AL, Travis WD, Zakowski MF, Kris MG, Ladanyi M (2012). Clarifying the spectrum of driver oncogene mutations in biomarker-verified squamous carcinoma of lung: lack of EGFR/KRAS and presence of PIK3CA/AKT1 mutations. Clinical cancer research.

[22] Pan Y, Wang R, Ye T, Li C, Hu H, Yu Y, Zhang Y, Wang L, Luo X, Li H, Li Y, Shen L, Sun Y (2014). Comprehensive analysis of oncogenic mutations in lung squamous cell carcinoma with minor glandular component. Chest.

[23] Zhang Q, Zhu L, Zhang J (2015). Epidermal growth factor receptor gene mutation status in pure squamous-cell lung cancer in Chinese patients. BMC cancer.

[24] Kang SM, Kang HJ, Shin JH, Kim H, Shin DH, Kim SK, Kim J-H, Chung KY, Kim SK, Chang J (2007). Identical epidermal growth factor receptor mutations in adenocarcinomatous and squamous cell carcinomatous components of adenosquamous carcinoma of the lung. Cancer.

[25] de Bruin EC, McGranahan N, Mitter R, Salm M, Wedge DC, Yates L, Jamal-Hanjani M, Shafi S, Murugaesu N, Rowan AJ, Gronroos E, Muhammad MA, Horswell S (2014). Spatial and temporal diversity in genomic instability processes defines lung cancer evolution. Science.

[26] Zhang J, Fujimoto J, Zhang J, Wedge DC, Song X, Zhang J, Seth S, Chow CW, Cao Y, Gumbs C, Gold KA, Kalhor N, Little L (2014). Intratumor heterogeneity in localized lung adenocarcinomas delineated by multiregion sequencing. Science.

